# Concomitant Graves' Disease and Ulcerative Colitis

**DOI:** 10.7759/cureus.78748

**Published:** 2025-02-08

**Authors:** Paige Webeler, Pardis Abdollahi Zarandi, Mani Bashyam

**Affiliations:** 1 Graduate Medical Education, Baylor Scott & White All Saints Medical Center, Fort Worth, USA; 2 Endocrinology and Diabetes, Baylor Scott & White Medical Center, Grapevine, USA

**Keywords:** autoimmune disease, graves´disease, hyperthyroid, methimazole, ulcerative colitis (uc)

## Abstract

It is widely recognized that one autoimmune disorder is sometimes associated with an increased frequency of having another autoimmune condition. However, the simultaneous presentation of Ulcerative colitis and Graves’ disease is an underreported occurrence in the medical literature. We present the case of a previously healthy 36-year-old female who presented with acute symptoms of ulcerative colitis and Graves' disease simultaneously, leading to the diagnosis of both conditions within a short time frame. These diagnoses were supported by colonoscopy findings of diffuse colonic inflammation along with low thyroid-stimulating hormone and elevated thyroxine levels. Her symptoms of both Graves' disease and ulcerative colitis rapidly resolved with treatment of Graves’ disease. This case highlights the significance of developing a broad differential and considering the coexistence of multiple autoimmune conditions when systemic symptoms are present.

## Introduction

Ulcerative colitis (UC), a systemic condition marked with chronic inflammation of the bowel mucosa, is an autoimmune disease that often causes bloody diarrhea along with abdominal cramping. It can have a wide variety of extraintestinal clinical features including episcleritis, arthralgia, erythema nodosum, and hepatobiliary manifestations such as primary sclerosing cholangitis, and others [1]. It is thought that UC likely presents as a result of both genetic factors and environmental triggers. A first-degree relative of an individual with UC is four times more likely to also develop this disease. Interestingly, appendectomy offers the potential to provide protective effects against UC and the age at which it first appears [2]. 

Similarly, Graves’ disease (GD) is also a systemic autoimmune condition in which the body attacks the thyroid gland, causing it to become overactive in the production of thyroid hormone. This leads to characteristic symptoms of tremors, shaking, heat intolerance, diarrhea, and weight loss. Extrathyroid effects of GD include Graves’ eye ophthalmopathy and pretibial myxedema whereby the skin becomes thick and gains a coarse texture [3]. GD is more commonly diagnosed in females and individuals over the age of 30.

Both diseases are fairly common, affecting approximately 1% of the adult population in the United States [3,4]. These two diseases can have devastating effects on the quality of life of the individual afflicted with them. Since both of these disorders are autoimmune in nature it follows that they would occur together. However, the simultaneous onset and coexistence of GD and UC has been a rare observation in the literature. This case report presents a young female patient diagnosed with both UC and GD in a short time span, highlighting the rare simultaneous onset of these autoimmune disorders. This case underscores the importance of considering coexisting autoimmune conditions in patients with complex, multi-system presentations. 

## Case presentation

We present the case of a 36-year-old South Asian female patient with a past medical history of hypercholesterolemia who presented to our facility with complaints of acute hematochezia, nausea, non-bloody vomiting, and generalized abdominal pain that began 10 days prior to arrival. A review of systems also revealed unintentional weight loss of approximately 12 pounds since the onset of her symptoms. She denied recent travels outside of the city, changes to diet, consumption of raw or unpasteurized food, recent medication use, or contact with sick individuals. She also denied any history of similar symptoms and denied any thyroid problems in the past. Her surgical and family history were non-contributory. She denies consumption of alcohol or tobacco products. 

Upon arrival at the emergency department, she was afebrile and tachycardic with a heart rate of 123 beats per minute. Her respiratory rate was 17 breaths per minute, blood pressure 116/74 mmHg, and oxygen saturation 100% on room air. She met sepsis criteria given the occurrence of tachycardia and leukocytosis, the source likely originating from the gastrointestinal tract. Two sets of blood cultures were obtained and remained negative. She was admitted for sepsis likely due to gastrointestinal origin and hematochezia with associated anemia. Significant laboratory findings also include an elevated erythrocyte sedimentation rate (ESR) and stool calprotectin (Table 1). Comprehensive stool pathogen by PCR was performed which detected enteroaggregative *Escherichia coli*. She was treated with piperacillin-tazobactam and intravenous fluid therapy. Gastroenterology (GI) and Infectious Disease (ID) teams were consulted. The GI team did not recommend colonoscopy at this juncture given that her symptoms were improving with antibiotics and supportive care. The ID team recommended continuation of IV fluids and IV piperacillin-tazobactam 3.375 g for three days and a day course of ciprofloxacin 500 twice daily. 

**Table 1:** Lab values for first hospitalization

Lab investigation	Value	Units	Reference Value
White blood cells	14.9	x10^9/L	4.5-11
Hemoglobin	11.8	g/dL	14-18
Erythrocyte sedimentation rate (ESR)	83	mm/hr	0-20
Fecal calprotectin	1340	ug/g	0-50
Thyroid stimulating hormone (TSH)	<0.005	mIU/L	0.27-4.20
Free thyroxine (T4)	4.94	ng/dL	0.93-1.70
Free triiodothyronine (T3)	8.4	ng/dL	2.0-4.4
Thyrotropin Receptor Antibody (TRAB)	10.2	UI/L	0-1.75

Throughout her hospital course, she was noted to have multiple episodes of tachycardia with a range of 105-149 beats/minute. ECG rhythms revealed normal rhythm, transthoracic echocardiogram was unremarkable with an ejection fraction of 55-60%. Labs were acquired to assess for hyperthyroid and showed low thyroid-stimulating hormone (TSH) and elevated thyroxine (T4) (Table 1). She was not prescribed methimazole or any other thyroid medications. 

She was discharged on day four with ciprofloxacin 500 mg twice daily for three days. She followed up with her primary care provider who prescribed propranolol 10 mg three times daily for hyperthyroid-related tachycardia. 

**Figure 1:**
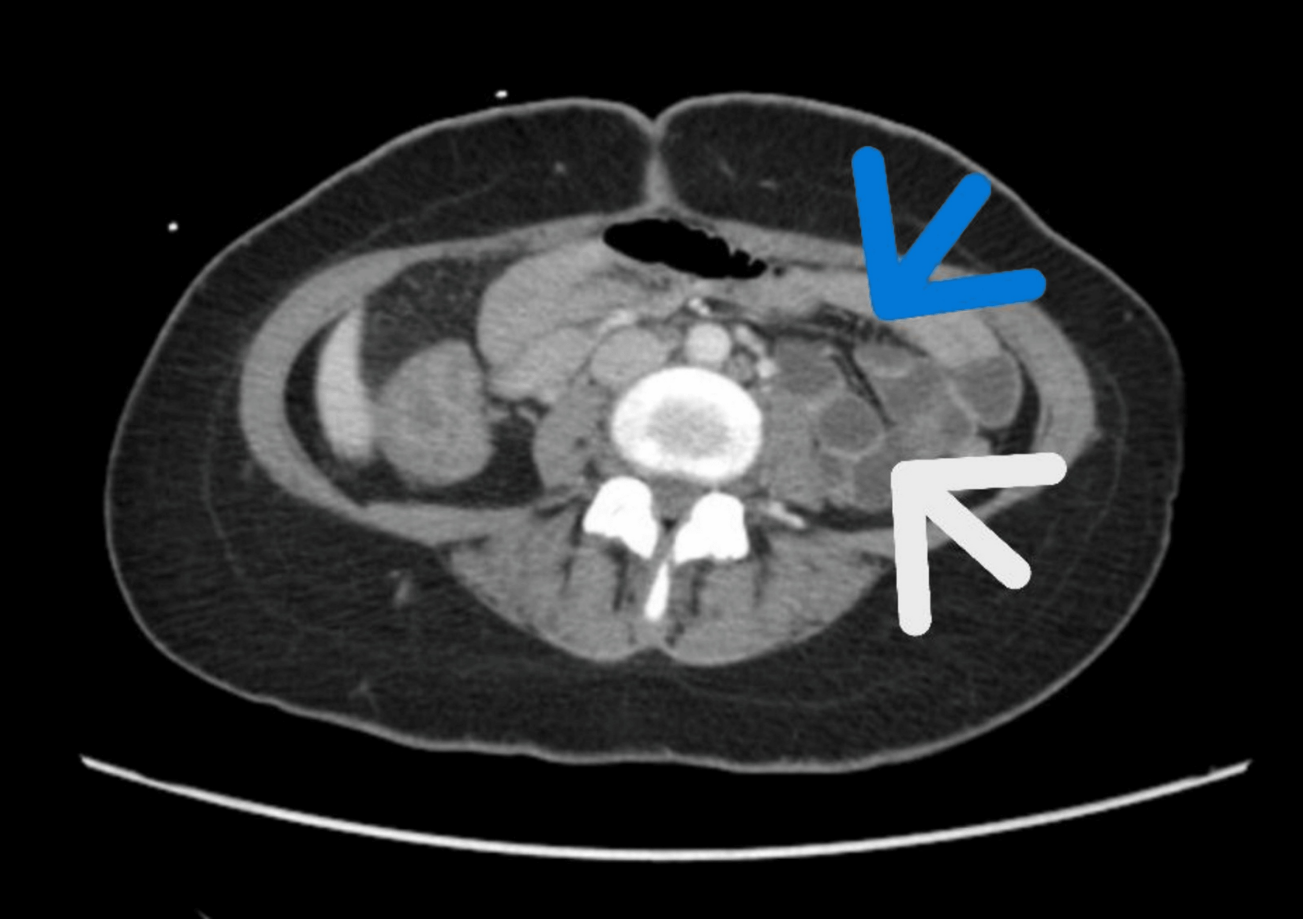
CT Abdomen/Pelvis: Diffuse inflammatory changes of the colon Blue arrow points to fat stranding. White arrow points to wall thickening.

Around 13 days after discharge, she returned to the Emergency Room complaining of four days of bloody diarrhea. She reported she had about 10 episodes of diarrhea daily. She also states she had generalized abdominal cramping, subjective fever, and palpitations. Initial vital signs were unremarkable apart from tachycardia of 123 beats/minute. Her thyroid gland was enlarged and non-tender to palpation. Labs were significant for normal WBC count, elevated ESR and stool calprotectin. TSH and Free T4 indicated worsening hyperthyroid (Table 2). The stool sample was negative for *Clostridium difficile,* *Salmonella*, Shiga toxin, *Shigella*, *Campylobacter*, and *Escherichia coli*. Contrast-enhanced computed tomography (CT) of the abdomen and pelvis showed diffuse thickening of the colonic wall without focal fluid collection (Figure 1). She was given ceftriaxone 1 g for four days. 

**Table 2:** Lab values for second admission

Lab Investigation	Value	Units	Reference Value
White blood cells	7.0	x10^9/L	4.5-11
Hemoglobin	11.2	g/dL	14-18
Erythrocyte sedimentation rate (ESR)	88	mm/hr	0-20
Fecal calprotectin	784	ug/g	0-50
Thyroid stimulating hormone (TSH)	<0.005	mIU/L	0.27-4.20
Free thyroxine (T4)	6.05	ng/dL	0.93-1.70

**Figure 2:**
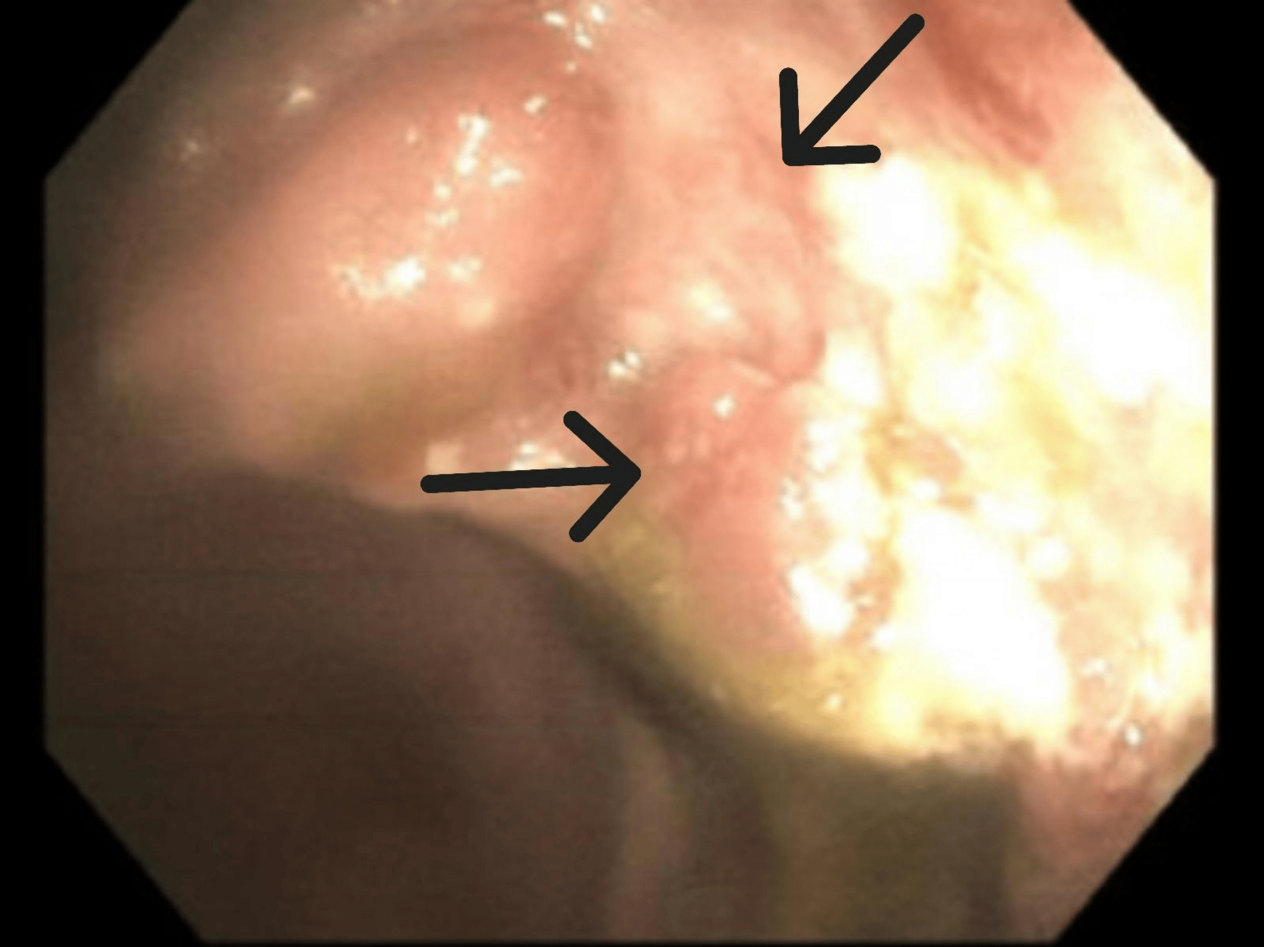
Colonoscopy image of cecum showing continuous mucosal inflammation Arrows indicate areas of significant mucosal edema and erythema.

**Figure 3:**
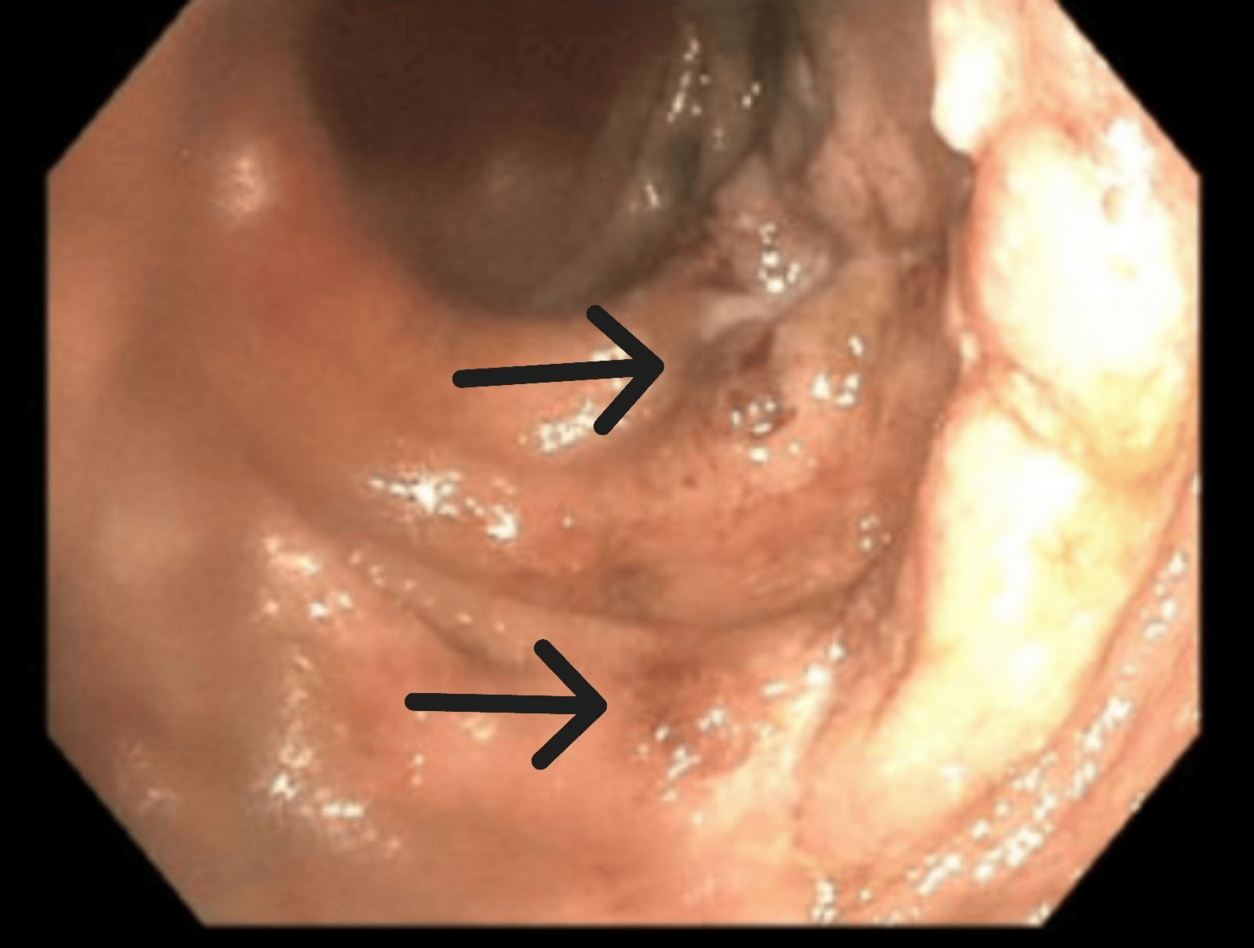
Colonoscopy image of ascending colon consistent with active inflammation Arrows indicate areas of mucosal erosion and bleeding.

**Figure 4:**
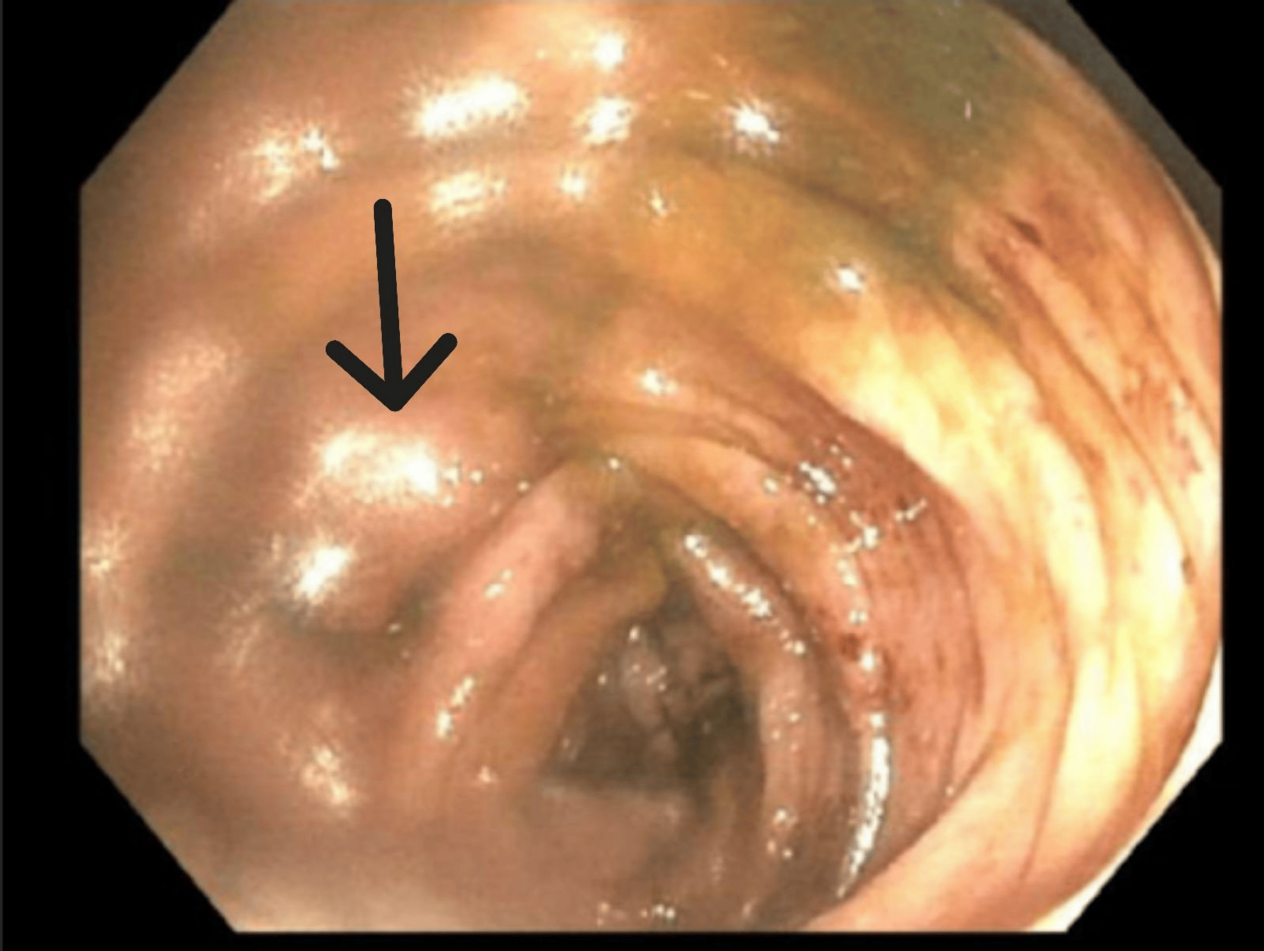
Colonoscopy image of descending colon shows diffusely edematous mucosa consistent with colitis Arrow shows area of significant edema and friability.

**Figure 5:**
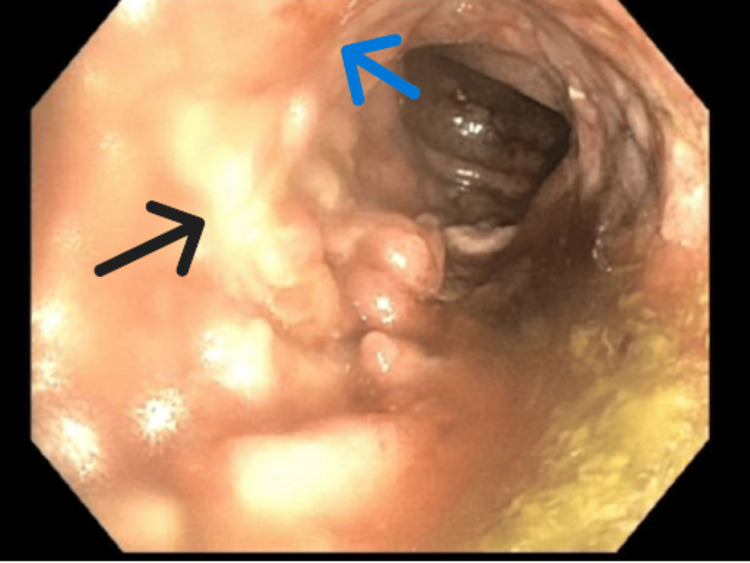
Colonoscopy image of sigmoid colon showing diffuse inflammatory changes Black arrow points to area of scarring and loss of vascular pattern. Blue arrow points to area of erythema and erosion.

**Figure 6:**
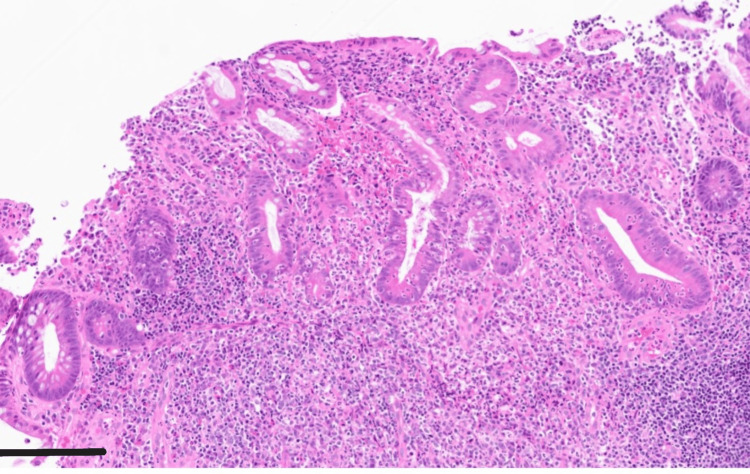
Pathology slide from colonoscopy biopsy This 40x magnification slide of the sigmoid colon uses Hematoxylin & eosin (H&E) staining. Scale bar indicates 100 micrometers. Biopsy shows generalized inflammatory cell infiltration consistent with severe active colitis.

The patient underwent a colonoscopy on the third day that showed pancolitis with a continuous and circumferential area of inflammation from the cecum to the rectum consistent with UC (Figures 2-5). The endoscopic findings were graded as Mayo score 3 which reflected severe ulcerations with spontaneous bleeding. Biopsies were taken from various segments of the colon and the pathological interpretation showed chronic inflammation with lymphoid follicles, consistent with inflammatory bowel disease (Figure 6). She was started on methylprednisolone 30 mg twice daily for three days. 

Due to persistent tachycardia and the recent diagnosis of hyperthyroidism secondary to GD, endocrinology was consulted who started the patient on methimazole 20 mg daily. The patient was discharged after five days of hospitalization with instructions to continue taking methimazole 20 mg daily, propranolol 10 mg daily, and prednisone 40 mg daily.

During her follow-up appointment one month after hospitalization, her TSH and T4 were notably improved (Table 3). The patient's symptoms of palpitations and diarrhea had improved and the dose of methimazole was decreased to 10 mg daily. The propranolol was then stopped and she was instructed to wean off of the prednisone taper. At the time of follow-up, no plans were made to start biologics due to the improvement in her diarrhea. 

**Table 3:** Lab values at one month follow-up appointment compared to second hospitalization

Lab Investigation	Value 2nd Hospitalization	Value at Follow Up Appointment	Units	Reference Value
Thyroid stimulating hormone (TSH)	<0.005	0.01	mIU/L	0.27-4.20
Free thyroxine (T4)	6.05	0.48	ng/dL	0.93-1.70

## Discussion

In this case report, we discuss the overlap of UC and GD. Though it is widely accepted that having one autoimmune disease predisposes to other autoimmune diseases, there are few reported cases of patients with both conditions. Though the exact cause is unknown, it is postulated to be from a dysregulation of the immune response to both environmental factors and the host itself [2]. 

UC is an inflammatory condition of the colon in which the body’s own innate and adaptive immune system attacks the colon. Cytokines and TH2 lymphocytes damage the colon, leading to painful, bloody diarrhea and abdominal pain [5]. This damaging host response extends beyond the colon to affect the joints, eyes, and skin. GD stems from antibodies produced by B cells that attack and inappropriately stimulate the thyroid receptor. TH2 cells also play a role in the attack of the thyroid gland. The stimulation by antibodies causes growth of the thyroid gland and unregulated production of thyroid hormone, leading to symptoms of weight loss, anxiety, and palpitations. GD also affects organs beyond the thyroid including the eyes and skin [6]. Since both of these diseases extend beyond their primary organ and utilize the TH2 response, it is feasible that the dysregulated immune response of one may affect or prompt the other.

The prevalence of GD and inflammatory bowel disease are each about 1% of the population [3,4]. Though there is no formally reported prevalence of the coexistence of these conditions, there are several case reports in the literature supporting their coexistence. One study suggested inflammatory bowel disease may increase the risk of GD by 24% [7]. There are cases reported of developing both UC and GD symptoms at the same time or of having one and then later developing the other. One such report is of a woman with a thyrotoxicosis episode from GD who developed UC immediately afterward [8]. Alternatively, a different case report shows a patient with known UC who presented to the hospital for flare and was found to have concomitant GD [9]. In another variation, a Japanese woman with UC developed GD years after she was first diagnosed with UC [10].

In our case, the patient presented with both new onset GD and UC. Literature reviews on this topic are limited due to the small number of case reports which highlights the importance of reporting cases and continuing to gather data to further evaluate this association. On her first admission our patient's symptoms were attributed to the positive *Escherichia coli* stool culture and her thyroid tests were not thought to be contributing to her clinical picture. When she re-presented with unresolved symptoms this led to a more comprehensive diagnostic workup which found the true cause of her symptoms. This emphasizes the importance of having a broad differential to avoid missing diagnoses. In her case, the diagnosis of one autoimmune condition, the UC, lead to further interest in the additional autoimmune condition, GD. Delayed diagnosis of either UC or GD can result in more inflammatory changes in the body. 

Treatment for UC involves a variety of biologics that target cytokines and immune factors along the immune pathway. These include agents against TNF alpha and products of TH2 induction such as IL-13 [5]. Treatment for GD includes methimazole which acts on thyroperoxidase, preventing the synthesis of thyroid hormone. Other options include surgery to remove the thyroid and radioactive iodine ablation [11]. In our case presentation, the patient was treated with methimazole with a resolution of her symptoms. Although at first glance her symptoms of diarrhea, tachycardia, and weight loss could have been solely attributed to UC, by looking into additional causes the GD was found and her symptoms were fully treated. One month after starting the methimazole she reported a resolution of palpitations and diarrhea and was regaining the weight she had lost. During her follow-up with GI, she was not started on biologics due to the drastic improvement in both her symptoms and the GD, which is thought to have triggered her symptoms. It follows that treating GD improved the symptoms of her UC and altered the course of her treatment in a positive way.

## Conclusions

UC and GD are autoimmune diseases that can both extend beyond their primary organ involvement and can present together, as in this case. It is thought that autoimmune diseases predispose to further autoimmune disease, although the exact cause is not known. Open-mindedness to this concept allowed our patient to be fully diagnosed and treated with a resolution of her symptoms, which underscores the importance of physician awareness of this phenomenon. More research is needed on the link between these disorders given the large number of people affected by each disease. Studies of how they are intertwined may help with early diagnosis and treatment. Though there are few reported cases, we add our case to the body of examples of concomitant GD and UC.
